# Socio-demographic disparities in basic under-two immunization coverage: insights from the 2016 Malawi demographic and health survey

**DOI:** 10.1186/s12889-025-22143-2

**Published:** 2025-03-05

**Authors:** Ololade Julius Baruwa, Monica Ewomazino Akokuwebe, Oluwafemi John Adeleye, Babatunde Makinde Gbadebo

**Affiliations:** 1 Centre for Social Sciences Research, University of Cape Town, Cape Town, Rondebosch, South Africa; 2 SAMRC Developmental Pathways for Health Research Unit, Department of Paediatrics, School of Clinical Medicine, Faculty of Health Sciences, University of the Witwatersrand, Johannesburg, South Africa; 3 Department of Epidemiology and Medical Statistics, Faculty of Public Health, College of Medicine, University of Ibadan, Ibadan, Nigeria

**Keywords:** Immunization, Vaccination, Malawi, Socio-demographic, Demographic and health survey

## Abstract

**Background:**

Childhood immunization is a vital component of public health, preventing the spread of infectious diseases and reducing child mortality. This study examines variations in basic immunization coverage across districts and explores socio-demographic disparities in immunization coverage among children aged 12–23 months in Malawi.

**Methods:**

The study employed a cross-sectional design, utilizing data from the 2016 MDHS, a nationally representative survey. The analysis included 3,248 children aged 12–23 months. Socio-demographic variables, including the child’s sex, maternal age, marital status (currently married), education, place of residence, region, wealth status, and employment status, were analysed using multivariate logistic regression models and a choropleth map to assess variations in basic immunization coverage across all 28 districts in Malawi.

**Results:**

The results showed that only 77.1% of children aged 12–23 months received basic immunization. The findings also highlighted significant variations in immunization coverage across different socio-demographic groups and among the 28 districts of Malawi. The highest coverage rates, ranging from 84.9 to 90.7%, were observed in Mwanza and Chiradzulu districts, while the lowest rates, between 65.3% and 68.0%, were found in Ntchisi, Blantyre, and Machinga districts. Multivariable analysis further indicated that children whose mothers were from a high household wealth index (OR = 1.45, 95% CI = 1.15–1.82), residing in rural areas (OR = 1.55, 95% CI = 1.20–2.01), currently married (OR = 1.33, 95% CI = 1.10–1.61), and with secondary or higher education (OR = 1.57, 95% CI = 1.11–2.21) were more likely to receive basic immunization.

**Conclusion:**

The study highlights low coverage of basic immunization in some districts as well as socio-demographic disparities in under-two immunization coverage in Malawi, necessitating tailored interventions such as educational campaigns and region-specific strategies.

## Introduction

Immunization for children under two years old (0–23 months) has significantly improved childhood survival and health outcomes, preventing millions of deaths globally [1, 2]. It serves as a vital intervention for disease prevention and an effective strategy for controlling infectious diseases among newborns and older infants [3, 4]. However, over the past decade, childhood vaccination coverage has stagnated, with over 14 million children worldwide either unvaccinated or under-vaccinated [2–4]. In response, the World Health Organization (WHO) developed the Immunization Agenda 2030 (IA2030) as a global strategy to enhance vaccine coverage and ensure that no one is left behind.

Vaccination coverage among newborns and older infants is unevenly distributed across settings and populations [1, 2]. Measuring socio-demographic inequalities is critical for identifying and addressing gaps in vaccine coverage that affect specific sub-populations. While childhood immunization coverage has increased globally and regionally since 1980, disparities persist, with high-income countries achieving substantially higher and more equitable coverage rates than low-income counterparts [5–7]. Reducing these inequalities is crucial for achieving the United Nations Sustainable Development Goals (SDGs).

Studies show that 60% of the world’s unvaccinated children reside in low- or middle-income countries, including Nigeria, India, Pakistan, Indonesia, Ethiopia, the Philippines, the Democratic Republic of Congo, Brazil, Angola, and Vietnam [4, 8]. Socioeconomic status, urban versus rural residence, maternal education, and other demographic characteristics significantly influence vaccination coverage, particularly in low- and middle-income countries [9, 10]. Marginalized communities remain under-vaccinated and disproportionately unvaccinated [10]. Early childhood disparities in immunization can have long-term effects on health outcomes, making it imperative to address these gaps.

In Malawi, the Expanded Programme on Immunization (EPI) was established in 1979, introducing vaccines such as Bacillus Calmette-Guérin (BCG) for tuberculosis, diphtheria, tetanus, and pertussis (DTP), oral polio vaccine (OPV), and measles-containing vaccine (MCV) [11, 12]. Despite these efforts, only 73% of children aged 12–23 months were fully vaccinated in 2016, falling short of the Malawian government’s goal of over 80% coverage [13]. However, recent data on whether significant improvements have been made remain limited.

Several studies have cited poor access to immunization services, the need to reach vulnerable pregnant and nursing women in rural communities, and inadequate supervision of routine immunization activities as key barriers to vaccine uptake [14–17]. Additionally, the COVID-19 pandemic further disrupted routine immunization and sensitization campaigns, hindering progress toward IA2030 goals [18–20]. A sustained and comprehensive intervention is urgently needed to ensure equitable, effective, and resilient immunization services in Malawi.

To enhance vaccination uptake and implement effective intervention strategies, it is crucial to identify both enablers and barriers to immunization coverage. Numerous studies have highlighted individual, household, and community-level determinants, including maternal age, education, household income, prenatal care attendance, skilled birth attendance, place of residence, geographical setting, awareness of immunization benefits, proximity to immunization clinics, cultural factors, and parental awareness of children’s vaccinations [21–25]. However, most studies have focused on either full or incomplete immunization, and no study has comprehensively examined variations in basic immunization coverage across Malawi’s districts or socio-demographic disparities in immunization among children aged 12–23 months.

This study aims to analyze socio-demographic disparities in basic immunization coverage among children aged 12–23 months in Malawi. Specifically, it investigates variations in coverage across districts and explores disparities based on socio-demographic factors. The findings will provide valuable insights for policymakers and health professionals, supporting the development of targeted interventions to improve childhood vaccination uptake and overall immunization coverage in Malawi.

## Materials and methods

### Study setting

Malawi is a low-income country with a population of approximately 20.4 million people, nearly 85% of whom reside in rural areas [4, 7]. Childhood vaccination is administered through local health facilities, either at fixed clinics or mobile outreach programs conducted by Health Surveillance Assistants (HSAs), who provide coverage for every village in Malawi [2, 11]. Vaccination services are free of charge. However, Malawi faces a significant shortage of healthcare personnel, often resulting in one health worker attending to a large number of infants seeking vaccination. This shortage can lead to inadequate documentation and follow-up, particularly in rural areas, where vaccines may not be administered on time or at all. Additionally, major stock-outs of Bacillus Calmette-Guérin (BCG) and diphtheria, pertussis, and tetanus (DPT) vaccines were reported at the central level in 2007 [26, 27]. Despite rapid urbanization, Malawi remains largely rural, with nearly 85% of the population still living in rural areas [28]. In Malawi, urban areas are classified as the four major metropolitan cities **—** Lilongwe, Blantyre, Mzuzu, and Zomba—along with secondary cities, including townships and district centers. All other areas in the country are designated as rural.

### Data source

The data utilized in this research was sourced from the children (KR file) dataset of the 2015-16 Malawi Demographic and Health Survey (MDHS) [27]. The MDHS 2015/16 was implemented by the National Statistics Office (NSO) and the Community Health Science Unit (CHSU). The government of Malawi, the National AIDS Commission (NAC), the United Nations Population Fund (UNFPA), the United Nations Children’s Fund (UNICEF), and the United Kingdom’s Department for International Development (DFID), the Centre for Disease Control and Prevention (CDC), and the United States Agency for International Development (USAID) provided the funds. Technical assistance on the survey was provided by ICF Macro through the MEASURE DHS program, a USAID-funded project. Using a two-stage cluster sampling technique based on enumeration areas defined in Malawi’s 2008 census, the survey gathered data on 17,395 children under the age of five. After thorough data wrangling and cleaning, the final analysis for this study was conducted on a subset of 3,248 children aged 12–23 months. Detailed information on sample size estimation and sampling strategies is accessible in the complete MDHS published reports at https://www.dhsprogram.com/.

### Study outcome

The outcome variable in this study is basic child immunization. A child is considered basic immunization if they have received all the recommended vaccinations, according to the vaccination policy framework in Malawi [27]. Specifically, basic immunization includes one dose of BCG, three doses of DPT-HepB-Hib, three doses of oral polio vaccine (excluding the birth dose), two doses of rotavirus vaccine, three doses of pneumococcal vaccine, and one dose of measles vaccine [27]. Children who received all the recommended vaccinations were coded as “1” and categorized as “yes,” while those who missed at least one recommended vaccine were coded as “0” and categorized as “no.”

### Explanatory variables

In this study, socio-demographic factors were considered as the explanatory variables. These factors were considered because they had a statistically significant association with basic child immunization in previous studies [11, 12, 14, 29]. The explanatory variables include sex of the child (male, female), maternal age [15–34], maternal education (no education, primary, and secondary or higher), mother is currently married (no and yes), place of residence (urban and rural), region (northern, central and southern), maternal wealth status (poor, middle and rich), and maternal employment status (working and not working).

### Statistical analyses

This analysis includes all relevant factors to control for potential confounding, with variable selection guided by the literature for their theoretical and policy relevance. This approach ensures both rigorous control and alignment with existing research. To explore the variations in basic immunization coverage by districts and to investigate the socio-demographic disparities in basic immunization coverage among 12-to-23-month-old children in Malawi, we followed four analytic steps. In the first step, we used descriptive statistical methods to describe the sample characteristics of respondents (frequency and percentages) (Table 1). The second step involved conducting a bivariate analysis, examining the cross-tabulation of all explanatory variables against the outcome variable using the Chi-square test (Table 1).

**Fig. 1:**
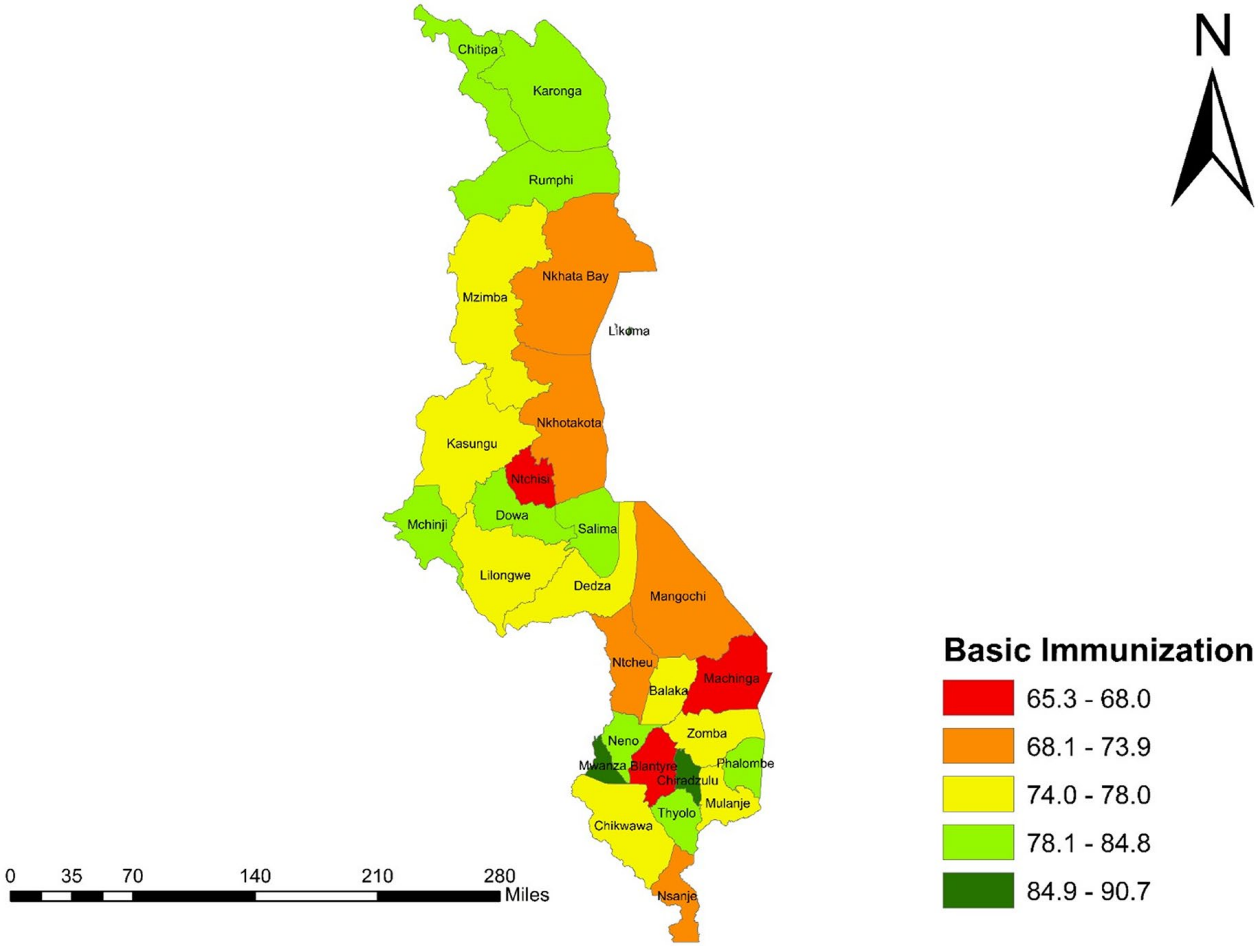
Choropleth map showing distribution of basic immunization coverage across districts in Malawi

In the third step, a spatial mapping analysis was conducted using ArcGIS Pro 2.8 software to determine district-specific proportions of basic immunization coverage in Malawi (Fig. 1). The spatial distribution of immunization prevalence was visualized through coloured density polygons, which depicted coverage by district. Spatial and spatiotemporal mapping methodologies are commonly used in public health research for their effectiveness in illustrating the distribution of health indicators, facility locations, and geographical accessibility. These methods also provide valuable insights into underserved areas and regions lacking sufficient healthcare interventions [26, 27, 30, 31]. For this analysis, the graduated symbol properties in ArcGIS were configured using a CSV file that contained the proportions of basic immunization coverage across Malawi’s districts. This data was integrated with the attribute table of the base map (shapefile), which included the x and y coordinates of each district’s polygon grid within Malawi’s geographical framework. The resulting analysis produced graduated colour maps representing basic immunization coverage for each district. Additionally, a coloured legend indicating varying proportions of immunization coverage was generated to enhance interpretability.

The fourth step involved using binary logistic regression to examine socio-demographic disparities in basic immunization coverage among children aged 12–23 months (Table 2). The analysis was performed in two stages (models). The first model assessed the independent relationship of each socio-demographic factor with immunization coverage, calculating unadjusted odds ratios (uOR) for each variable. The second model incorporated all the socio-demographic factors simultaneously to assess their combined influence on immunization coverage (adjusted odds ratios [aOR]). The binary logistic regression model was applied to investigate the predictive relationship between the dichotomous dependent variable, using the value 0 or 1 (i.e. failure or success), and a set of explanatory variables. Therefore, the binary logistic regression model is expressed as [32, 33]:

$$\:\mathbf{L}\mathbf{o}\mathbf{g}\:\left(\varvec{\uprho\:}/1-\:\varvec{\uprho\:}\right)\:=\mathbf{a}\:+\:\varvec{\Sigma\:}\:\varvec{\upbeta\:}\mathbf{i}\mathbf{{\rm\:X}}\mathbf{i}\:+\:\mathfrak{e,}$$

where $\:\varvec{\uprho\:}$ is the event of occurrence (outcome variable), $\:\mathbf{{\rm\:X}}\mathbf{i}$ is the explanatory variables, $\:\varvec{\upbeta\:}\mathbf{i}$ is the size of the coefficient of explanatory variables, and $\:\mathfrak{e}$ is the margin of error. For model assessment, the log-likelihood ratio (LLR) was employed and sample weight was applied to address related concerns of over-and under-sampling. However, the SVY command was used to account for the complex survey design and generalizability of the findings. The statistical significance for all tests was set at *ρ* < 0.05.

The data were analysed with STATA version 15.4 for MacOs, and three steps were pursued to analyze the data. All variables were weighted to guarantee the representativeness of the survey data and account for the complex sampling strategy used in the survey.

## Results

### Socio-demographic characteristics of the study population

Table 1 presents the characteristics of the study population. Findings revealed that 77.1% of children aged 12–23 months had received the basic immunization. About 50% (49.9%) of the children are male, and 31.9% are born to mothers who are between the ages of 20–24 years. The table further shows that 68.2% of the mothers have primary education, 45.9% are from poor households, 84.4% live in rural areas, 46.1% are from the Southern region, 46.1% are from the Southern region, 77.4% are currently married, and 64.9% are employed (Table 1).

### Prevalence and bivariate associations of basic immunization coverage among children aged 12–23 months by socio-demographic factors

Table 1 also presents the bivariate analysis. The bivariate analysis showed that education, wealth status, and marriage status have significant relationships with immunization coverage among children aged 12–23 months. Basic immunization coverage was higher among female children (78.5%) than male children (75.8%). Basic immunization coverage was also more prevalent among children born to mothers aged 30–34 years (79.3%) compared to those born to mothers aged 15–19 years (72.7%). The findings revealed that coverage of basic immunization was more pronounced among children whose mothers had secondary or higher education (82.7%) compared to those whose mothers had no formal education (74.1%).

The prevalence of basic immunization coverage was higher (80.9%) among children whose mothers are from rich households compared to those of poor and middle-wealth status (74.9% and 75.9%, respectively). Further, the prevalence of basic immunization was 77.5% among children whose mothers resided in rural areas, 79.6% among children whose mothers lived in the northern region, 78.4% among children whose mothers were currently married, and 77.9% among children whose mothers were working (Table 1).

### Choropleth map showing the basic immunization coverage among children aged 12–23 months across districts in Malawi

The distribution of basic immunization coverage across the 28 districts in Malawi is illustrated in Fig. 1 below. *High coverage* (≥ 90%) was observed in Mwanza and Chiradzulu districts, with proportions ranging from 84.9 to 90.7%. *Moderate coverage* (80-89%) was found in the districts of Karonga, Rumphi, Chitipa, Mchinji, Dowa, Salima, Thyolo, Neno, and Phalombe, with coverage ranging from 78.1 to 84.8%. *Low coverage* (< 80%) was recorded in Ntchisi, Blantyre, and Machinga districts, with proportions ranging from 65.3 to 68.0% (Fig. 1). This reclassification follows the WHO recommendations on immunization coverage, which categorizes high coverage as ≥ 90%, moderate coverage as 80-89%, and low coverage as < 80% [35].

### Multivariable analysis of factors associated with basic immunization coverage among children aged 12–23 months

Table 2 presents the odds ratio and confidence interval of the relationship between socio-demographic factors and basic immunization coverage among children aged 12–23 months in Malawi. The multivariable analysis showed that mothers’ educational level, household wealth status, and marital status (currently married) are protective of basic immunization coverage in the unadjusted and adjusted analyses. Maternal age showed a significant association with basic immunization coverage in the unadjusted analysis but not in the adjusted, while place of residence showed a significant association in the adjusted analysis but in the unadjusted. The multivariate analysis showed that the odds of basic immunization coverage among children aged 12–23 months increase with the level of the mother’s education. For instance, the adjusted logistic regression analysis showed that the likelihood of basic immunization coverage is 1.57 times higher among children whose mothers had secondary or higher education (aOR = 1.57; 95%CI = 1.11–2.21) compared to those with no formal education. Likewise, the odds of basic immunization coverage were 1.45 times higher among children born to mothers from rich households (aOR = 1.45; 95%CI = 1.15–1.82) compared to their counterparts from poor households (Table 2). The likelihood of basic immunization coverage was 1.55 times higher among children whose mothers reside in a rural area (aOR = 1.55; 95%CI = 1.20–2.01) compared to those whose mothers reside in an urban area. The odds of basic immunization coverage were 1.33 times higher among children whose mothers were currently married (aOR = 1.33; 95%CI = 1.10–1.61) compared to those whose mothers were currently unmarried (Table 2).

**Table 1:** Study participants’ characteristics and outcome and its Chi-Square association

	Total sample	No Basic Immunization	Basic Immunization	Chi-square (χ^2^) ρ-value
** Variables**	N = 3248	22.9 (743)	77.1 (2505)	
**Sex of Child**	**% (n)**	**% (n)**	**% (n)**	
Male	49.9 (1622)	24.2 (393)	75.8 (1229)	0.067
Female	50.1 (1626)	21.5 (350)	78.5 (1276)	
**Maternal age**				
15–19	10.4 (337)	27.3 (92)	72.7 (245)	0.115
20–24	31.9 (1036)	23.5 (243)	76.5 (793)	
25–29	22.7 (738)	21.0 (155)	79.0 (583)	
30–34	18.3 (595)	20.7 (123)	79.3 (472)	
35 and above	16.7 (542)	24.0 (130)	76.0 (412)	
**Education**				
No education	10.9 (355)	25.9 (92)	74.1 (263)	< 0.001*
Primary	68.2 (2216)	24.1 (534)	75.9 (1682)	
Secondary/Higher	20.8 (677)	17.3 (117)	82.7 (560)	
**Wealth Status**				
Poor	45.9 (1491)	25.2 (375)	74.9 (1116)	< 0.001*
Middle	19.8 (643)	24.1 (155)	75.9 (488)	
Rich	34.3 (1114)	19.1 (213)	80.9 (901)	
**Place of Residence**				
Urban	15.6 (508)	24.8 (126)	75.2 (382)	0.260
Rural	84.4 (2740)	22.5 (617)	77.5 (2123)	
**Region**				
Northern region	18.6 (603)	20.4 (123)	79.6 (480)	0.274
Central region	35.3 (1147)	23.5 (270)	76.5 (877)	
Southern region	46.1 (1498)	23.4 (350)	76.6 (1148)	
**Currently Married**				
No	22.6 (733)	27.2 (199)	72.9 (534)	< 0.001*
Yes	77.4 (2515)	21.6 (544)	78.4 (1971)	
**Employment Status**				
Not Working	35.1 (1141)	24.4 (278)	75.6 (863)	0.137
Working	64.9 (2107)	22.1 (465)	77.9 (1642)	

**Source**: Authors’ compilation; * - Significant at 5%

**Table 2:** Logistic regression model showing the association between socio-demographic factors and under-two (12-23 months) basic immunization coverage in Malawi

Variables	uOR (95% C.I)	aOR (95% C.I)
**Sex of Child**		
Male	1	1
Female	1.17 (0.99–1.37)	1.15 (0.98–1.36)
**Maternal age**		
15–19	1	1
20–24	1.23 (0.93–1.62)	1.09 (0.82–1.45)
25–29	1.41 (1.05–1.9)**	1.21 (0.89–1.65)
30–34	1.44 (1.06–1.97)**	1.27 (0.93–1.75)
35 and above	1.19 (0.87–1.62)	1.09 (0.78–1.51)
**Education**		
No education	1	1
Primary	1.1 (0.85–1.42)	1.08 (0.83–1.42)
Secondary/Higher	1.67 (1.23–2.28)***	1.57 (1.11–2.21)**
**Wealth Status**		
Poor	1	1
Middle	1.06 (0.85–1.31)	1.02 (0.82–1.27)
Rich	1.42 (1.18–1.72)***	1.45 (1.15–1.82)***
**Place of Residence**		
Urban	1	1
Rural	1.14 (0.91–1.42)	1.55 (1.2–2.01)***
**Region**		
Northern region	1	1
Central region	0.83 (0.65–1.06)	0.93 (0.72–1.19)
Southern region	0.84 (0.67–1.06)	0.96 (0.76–1.22)
**Currently Married**		
No	1	1
Yes	1.35 (1.12–1.63)***	1.33 (1.10–1.61)***
**Employment Status**		
Not Working	1	1
Working	1.14 (0.96–1.35)	1.13 (0.95–1.35)

* *p* < 0.1, ** *p* < 0.05, *** *p* < 0.01; uOR – Unadjusted Odds Ratio; aOR – Adjusted Odds Ratio; C.I – Confidence Interval

## Discussion

This study explores socio-demographic disparities in basic immunization coverage among children aged 12–23 months in Malawi. Our findings indicate that only 77.1% of these children had received basic immunization, closely aligning with a previous study that reported 76% coverage in the same age group [28]. This coverage rate falls short of the World Health Organization’s (WHO) recommended 90% immunization coverage benchmark, highlighting a significant gap in achieving optimal vaccination rates for this vulnerable population. The low immunization rates pose a potential public health risk, underscoring the need for targeted interventions and awareness campaigns. Healthcare policymakers and practitioners must collaborate to develop strategies that enhance immunization coverage for infants and toddlers, thereby safeguarding their health and contributing to overall community well-being.

Our study reveals significant disparities in basic immunization coverage across Malawi’s 28 districts. Mwanza and Chiradzulu recorded the highest immunization rates, while Ntchisi, Blantyre, and Machinga exhibited the lowest. These variations highlight the importance of exploring district-specific factors that influence immunization uptake. The high coverage observed in Mwanza and Chiradzulu may be attributed to several factors, including proximity to healthcare facilities, which plays a crucial role in vaccine accessibility. Residing closer to vaccination services has been linked to increased immunization rates, whereas longer travel distances often hinder access and reduce coverage. Conversely, districts such as Ntchisi, Blantyre, and Machinga, which reported lower immunization rates, may face challenges such as limited access to healthcare services, lower socioeconomic conditions, and inadequate health education [36]. Addressing these barriers is essential for improving immunization coverage. To enhance immunization rates in underperforming districts, targeted interventions are required. These may include strengthening healthcare infrastructure to minimize travel distances and addressing socioeconomic barriers that restrict healthcare access. By drawing lessons from districts with high coverage and implementing localized strategies, Malawi can work toward achieving equitable immunization coverage across all regions.

Our multivariable analysis found a significant association between maternal age and basic immunization coverage. Specifically, the likelihood of completing basic immunization schedules increased with maternal age, a finding that aligns with previous studies [28, 34]. Older mothers may have socio-economic advantages and better health literacy, enabling them to engage more effectively with healthcare systems. These advantages include a greater understanding of immunization’s importance, increased autonomy in decision-making, and improved access to healthcare facilities.

Consistent with previous studies [28, 34], our findings indicate that children of educated mothers are more likely than those of non-educated mothers to receive the basic under-two immunization. The possible explanation is that maternal education enhance health literacy, allowing mothers to recognize the importance of immunizing their children [14, 37]. Educated mothers are also more capable of navigating healthcare systems, ensuring they attend immunization clinics on time and adhere to vaccination schedules. Furthermore, through exposure to health education programmes and media, educated mothers may possess greater knowledge about preventive healthcare practices, such as immunization. A positive attitude toward vaccination and a proactive approach to making sure their children receive their vaccinations on time can be attributed to this increased awareness [38].

An interesting finding of this study is that children born to mothers in rural settings have a higher likelihood of receiving basic under-two immunization compared to those in urban areas. This finding contradicts previous studies [37, 40]. However, this observed pattern could be the result of several factors. First, due to the possible lack of access to centralized healthcare facilities, community-based healthcare interventions, such as immunization programmes, may be given more importance in rural areas. Hence, community networks that promote collective participation in preventive health practices and facilitate the dissemination of health information may be beneficial to mothers living in rural areas. Second, people living in rural areas may have a greater understanding of the value of immunization as a preventative measure against common diseases that are common in these areas, as these populations frequently face unique health challenges [39–41]. Third, rural communities’ geographic dispersion may also lead to focused outreach initiatives that bring healthcare services, such as immunization, closer to families. However, it is important to consider that Malawi’s population is predominantly rural, which may influence this association.

The multivariable analysis indicating that children born to mothers from rich households were more likely to receive basic under-two immunization aligns with a previous study [40, 41]. Higher socioeconomic status, as reflected in the rich household wealth index, often provides families with the financial capacity to cover transportation costs, purchase health insurance, and manage other expenses related to healthcare services, including immunization. Additionally, economic stability in richer households may contribute to better health literacy, heightened awareness of the importance of immunization, and a stronger ability to prioritize healthcare needs. Rich households are more likely to have access to information, resources, and opportunities that enable them to utilize health services more effectively, ensuring their children receive timely vaccinations.

Finally, the finding that the odds of basic immunization coverage were higher among children whose mothers were currently married compared to those whose mothers were unmarried suggests that marital status plays a critical role in healthcare-seeking behaviour and child health outcomes, including immunization. Married mothers may benefit from greater social and emotional support from their spouses, family members, or the broader community [24, 41]. This support can make it easier to prioritize healthcare needs like immunization, especially in low-resource settings where logistical and financial barriers exist. Spouses may assist with household responsibilities, childcare, or transport to healthcare facilities, thereby reducing the burden on mothers and enabling them to adhere to vaccination schedules.

### Strengths and limitations

Our study highlights socio-demographic disparities in basic immunization coverage among children aged 12–23 months in Malawi, utilizing nationally representative data from the 2016 Demographic and Health Survey (DHS). While our findings provide valuable insights into factors influencing immunization coverage, it is essential to acknowledge the study’s limitations. First, recall bias may affect the accuracy of immunization data, as mothers might not always accurately recall their children’s vaccination history. Second, the cross-sectional nature of the data limits our ability to establish causal relationships, meaning observed associations should be interpreted with caution. Additionally, while the DHS provides robust data, it may not capture all potential determinants of immunization coverage, such as healthcare accessibility and cultural influences on immunization. Despite these limitations, our study contributes to the existing body of knowledge by identifying key socio-demographic disparities in immunization coverage. These findings can inform future research and targeted interventions aimed at reducing inequities and improving vaccination rates among young children in Malawi.

## Conclusions

This study highlights the need to address socio-demographic disparities in basic immunization coverage among children aged 12–23 months in Malawi, where coverage remains below the WHO’s recommended 90% target. The findings suggest that maternal age, education, wealth status, marital status, and rural residence are associated with basic immunization, underscoring the importance of targeted interventions to improve healthcare access and awareness. Notably, children from rural areas, wealthier households, and those with educated or married mothers had higher basic immunization coverage. However, variations in coverage across districts and socio-economic groups highlight inequities that require policy intervention.

Efforts should focus on expanding access in underserved districts and urban settings and supporting mothers through socio-economic initiatives. Addressing systemic barriers to healthcare can help ensure more equitable immunization opportunities for all children, regardless of geographic or socio-economic background. Collaborative approaches involving policymakers, healthcare providers, and community stakeholders will be key to closing the immunization gap and advancing global health goals.

## Data Availability

The study used secondary data from dhsprogram.org, which is publicly available at https:/ /dhsprogram.com/data/available-datasets.cfm.
